# Novel radiomics evaluation of bone formation utilizing multimodal (SPECT/X-ray CT) *in vivo* imaging

**DOI:** 10.1371/journal.pone.0204423

**Published:** 2018-09-25

**Authors:** Ferenc Budán, Krisztián Szigeti, Miklós Weszl, Ildikó Horváth, Erika Balogh, Reem Kanaan, Károly Berényi, Zsombor Lacza, Domokos Máthé, Zoltán Gyöngyi

**Affiliations:** 1 Department of Public Health Medicine, Medical School, University of Pécs, Pécs, Hungary; 2 MedProDevelop, Pécs, Hungary; 3 Department of Biophysics and Radiation Biology, Semmelweis University, Budapest, Hungary; 4 Department of Health Economics, Corvinus University of Budapest, Budapest, Hungary; 5 Institute of Clinical Experimental Research, Semmelweis University, Budapest, Hungary; 6 CROmed Translational Research Centers, Budapest, Hungary; Lee Kong Chian School of Medicine, SINGAPORE

## Abstract

Although an extensive research is being undertaken, the ideal bone graft and evaluation method of the bone formation draw still a warranted attention. The purpose of this study was to develop a novel multimodal radiomics evaluation method, utilizing X-ray computed tomography (CT) and single photon emission computed tomography (SPECT) with Tc-99m-Methyl diphosphonate (Tc-99m-MDP) tracer. These modalities are intended to provide quantitative data concerning the mineral bone density (after evaluation it is referred to as opacity) and the osteoblast activity, at the same time. The properties of bone formation process within poly (methyl methacrylate)-based bone cement graft (PMMA) was compared to that of albumin coated, sterilized, antigen-extracted freeze-dried human bone grafts (HLBC), in caudal vertebrae (C5) of rats. The animals were scanned at 3 and 8 weeks after surgery. In both groups, the mean opacity increased, while the mean Tc-99m-MDP activity decreased. The later parameter was significant (n = 4, p = 0.002) only in HLBC group. The linear regression analysis of PMMA-treated group variables (mean opacity increase; mean Tc-99m-MDP activity decrease), revealed a negative correlation with the medium strength (r = 0.395, p = 0.605). Whereas, it showed strong positive correlation when HLBC group variables were analyzed (r = 0.772, p = 0.012). These results indicate that using HLBC grafts is advantageous in terms of the osteoblast activity and bone vascularization over PMMA cement. Using this regression analysis method, we were able to distinguish characteristics that otherwise could not be distinguished by a regular data analysis. Hence, we propose utilizing this novel method in preclinical tests, and in clinical monitoring of bone healing, in order to improve diagnosis of bone-related diseases.

## Introduction

Bone grafting is the replacement of missing bone utilizing a surgical procedure. The ideal bone grafts possess certain essential properties, such as efficacious and safe use, biocompatibility, appropriate mechanical/chemical attributions, cost-effectiveness, convenient usage, as well as production and availability in a large quantity [1]. Materials, such as poly (methyl methacrylate)-based (PMMA) cements were widely used. Extensive research is going on in animals to detect the safety of newly developed experimental bone graft materials [2]. Clinical studies are also ongoing so as to map the beneficial effects of novel bone grafts. Adding or mixing several materials such as demineralized bone matrix (DBM), ceramics, coral, graft composites, and bone morphogenetic proteins, etc. to the bone cement can improve its biological function [3–5]. Biomaterials, like albumin coated, sterilized, antigen-extracted freeze-dried human bone graft (HLBC), can acquire function via chemical treatments, for example by coating or surface grafting. These modifications improve wound healing or even control cell fate *in situ* [6]. However, ideal bone grafts are still necessary [7]. Furthermore, suitable evaluation methods of specific bone grafts are yet to be developed.

The rat tail model has major advantages over other animal models. These include effectiveness regarding to its cost as well as the accessibility of the bones [8–10]. However, rat bone structure lacks a haversian system [11]. Apart from the bone remodeling process, microstructure of rat bone is similar to more advanced species, with regard to bone formation properties [12]. These properties depend mainly on the osteoblast activity and bone vascularization [13].

Consequently, the bone-healing process could be followed up, utilizing a rat tail model, in order to evaluate the synthetically modified and the biologically derived bone substitutes as well as the xenogeneic bone graft [9, 14].

The X-ray attenuation of a specific voxel correlates to the cube of effective atomic numbers of the components. These effective atomic numbers contribute to the attenuation according to their molar quantity [15, 16].

Thus, the X-ray CT as a noninvasive technique is especially well-suited for applications involving the measurements of bone density, owing to the high signal contrast between bone and soft tissue [16]. The X-ray attenuation (after specific data processing, see below) in this context is referred to as opacity. Moreover, the specific technical properties of the utilized CT (e.g. beam-energy profiles), as well as other factors, can influence the measurements [16]. Therefore, the mean opacity values of the examined region of interest (ROI) can be normalized to the opacity values of an intact ROI of the same object. The same is true of the adequate selection of X-ray attenuation “density” cut-offs in order to filter-out the tissues (or objects), irrelevant from perspective of examinations [17].

In the clinical practice, bone scintigraphy is a widespread screening method that is based on osteoblast labeling by Tc-99m-Methyl diphosphonate (Tc-99m-MDP) [18]. The Tc-99m-MDP accumulates in the bone by chemical adsorption and incorporates into the hydroxyapatite structure [19]. Areas with high osteogenic activity are Tc-99m-MDP absorbers and can be identified with gamma camera [19]. SPECT imaging with a Tc-99m-MDP tracer has been used frequently in nuclear medicine for the *in vivo* diagnosis of abnormalities in bone formation and remodeling, including osteogenic tumors or metabolic bone diseases [18–20].

The multimodal *in vivo* imaging is capable to provide anatomical and functional information simultaneously [21]. This could be applied to obtain more quantified and comparable data. In previous study, the bone formation was investigated with multimodal NanoSPECT/CT in rat tail implant model [22]. The results have revealed that the bone formation is supposed to be detectable three weeks after bone graft insertion with both modalities [22]. Additionally, eight weeks following the bone graft insertion, the healing process might still be ongoing as indicated by the increase in the bone opacity along with the decrease of the standardized uptake volume (SUV) of Tc-99m-MDP [10, 22]. This healing process lasts 12–14 weeks following the surgery [10, 22].

Moreover, multiple testing with the same animal, combined with radiomics evaluation, provides more relevant biological information [10, 17].

The purpose of this study was to find a new radiomics evaluation method, calculating linear regression from the bone opacity and activity of Tc-99 m-MDP, regarding the bone healing properties. HLBC and PMMA were aimed to examine in this study. A further goal of this study, was to highlight that implementing a radiomics evaluation method, can result in reducing the number of animals needed for conducting experiments. This goal is in coherence with the European Union (EU) directive 2010/63/EU on the protection of animals [10, 23].

## Materials and methods

### Animal model

Two groups (n = 5) of female Wistar rats (Crl(Wi)Br, Charles River; 650–950 g from the breeding colony of Semmelweis University (Hungary)) were kept in light controlled, air-conditioned rooms and fed *ad libitum*. All the procedures were conducted in accordance with the ARRIVE guidelines and the guidelines set by the European Communities Council Directive (86/609 EEC) and approved by the Animal Care and Use Committee of Semmelweis University (protocol number: XIV-I-001/29-7/2012).

The surgical model of Blazsek et al. was applied, described briefly below [14]. In the spongious model of Blazsek et al., rats were anesthetized with sodium pentobarbital (Nembutal (Sigma-Aldrich) 40 mg/kg body weight (b.w.), by intraperitoneal (i.p.) injection). The tail above the C4 vertebrae was ligatured to control bleeding during surgery. The tail was disinfected, then was partly removed after the C5 vertebra. A 5–6 mm incision was made at the level of caudal *vertebrae* (C4–C5). The skin was retracted and the vertebras were exposed under sterile conditions. In the exposed central surface of C5, a 1 mm diameter and 5 mm deep hole was formed using an electric drill, corresponding to the size of a titanium screw. Subsequently, a hole was made (2.0 mm diameter and 3.5 mm depth) creating an “empty” cylinder, which allowed 360° rotation. Screw-type titanium implants (1.2 mm diameter and 3.5 mm length) were fabricated and their surface roughened using sandblasting (Full-Tech Company, Hungary). The sterilized screws were introduced into the 5 mm deep thin hole. Following insertion of the implant the skin was repositioned over the implant and tightly sutured. The surgical wound was protected aseptically by a plastic methyl-methacrylate butyl-acrylate butyl-methacrylate copolymer, diisooctyl phthalate film layer (Plastubol^®^, Pannonpharma Ltd. Hungary). The rats were kept in individual cages to insure appropriate hygiene and wound healing during the first two weeks following surgery. In each animal, 3 weeks after surgical intervention, the titanium implant was removed and the remained hole was filled with the experimental materials, see below [14].

In this study, one animal group (n = 5) were treated with chemically sterilized, antigen-extracted HLBC (West Hungarian Regional Tissue Bank) and one group (n = 5) with PMMA-based cement (Vertebroplastic, DePuy, USA) experimental materials. The HLBC was pretreated; a human serum albumin coating method (200g/1000ml, BIOTEST) was applied [24].

After the experiment, animals were killed by cervical dislocation. The autopsy was carried out in order to detect potential abnormalities e.g. inflammation.

### Detection and image evaluation

The animals (n = 5) were scanned 3 and 8 weeks after surgery using a quantitative multiplexed multipinhole NanoSPECT/CT+ (Mediso, Hungary). The acquisition time was 30 min for X-ray CT. The reconstructed cubic voxel side size was 150 μm in a 198 × 198 × 546 pixel matrix in both the SPECT and CT modalities. Fusion (Mediso Ltd., Hungary) and VivoQuant (inviCRO LLC, US) image analysis softwares were used to further analyze the reconstructed, reoriented and co-registered images by drawing appropriate volumes of interests (VOIs) over the specific caudal (C4, C5) *vertebrae*.

From the whole SPECT image, the C5 and C4 *vertebrae* of tail were selected then VOIs were marked. The isotope activity in VOI was summed. The radioactive dose concentration of Tc-99m-MDP was determined by dividing measured radioactivity in an animal (in MBq) by the whole body weight (in grams) of the animal to calculate the standard uptake volume (SUV) [22].

The summarized absorbance of VOI was calculated. The voxels in VOI with attenuation below 1400 Hounsfield Unit (HU) were cut off in order to filter the soft tissue from total X-ray attenuation of interest vertebra. Thus, only the mineralized bone tissue of C5 and C4 *vertebrae* was evaluated. This attenuation values were normalized in the following manner. The ratio of summarized bone mineral density (attenuation) of treated and control *vertebrae* was calculated representing normalized bone mineral density (in this context is referred to as opacity). From each group, 1 experimental animal with signs of inflammation was removed. These calculations were performed for all animals also 3 and 8 weeks after bone graft insertion as well and the statistical mean and SD were calculated.

The same quantitative multiplexed multipinhole NanoSPECT/CT+ (Mediso, Hungary) was utilized for selected rats (n = 4) to carry out a SPECT examination 3 and 8 weeks after surgery. The rats were scanned 3 h after the injection of 80 MBq of Tc-99m-MDP. After the acquisition, the data were reconstructed with the HiSPECT software.

These results were normalized in the same way as mentioned before. Thus, SUV ratios were used to measure the treatment response less depended on noise and image resolution. Normalized SUV values in this context are referred to as Tc-99m-MDP activity. This process was performed on each animal. The mean and SD values of Tc-99m-MDP activity in specific PMMA and HLBC groups were calculated in both of the examined time periods.

2-tailed Student's t-test was performed for statistical evaluation of the mean and standard deviation (SD) values of bone opacity as well as the Tc-99m-MDP activity in both of the examined time periods. The ratios of mean parameters at three and at eight weeks after bone graft insertion were determined. The means of opacity change were expressed in percent of opacity increasing from third to the eighth week. The mean of Tc-99m-MDP activity change was calculated for both groups utilizing the mentioned ratios between the parameters of three and of eight weeks after bone graft insertion and expressed in the percent of activity decreasing from third week to the eighth week. Finally, a linear regression analysis was carried out to examine the correlation between the opacity change and the Tc-99m-MDP activity change.

## Results

**Fig 1:**
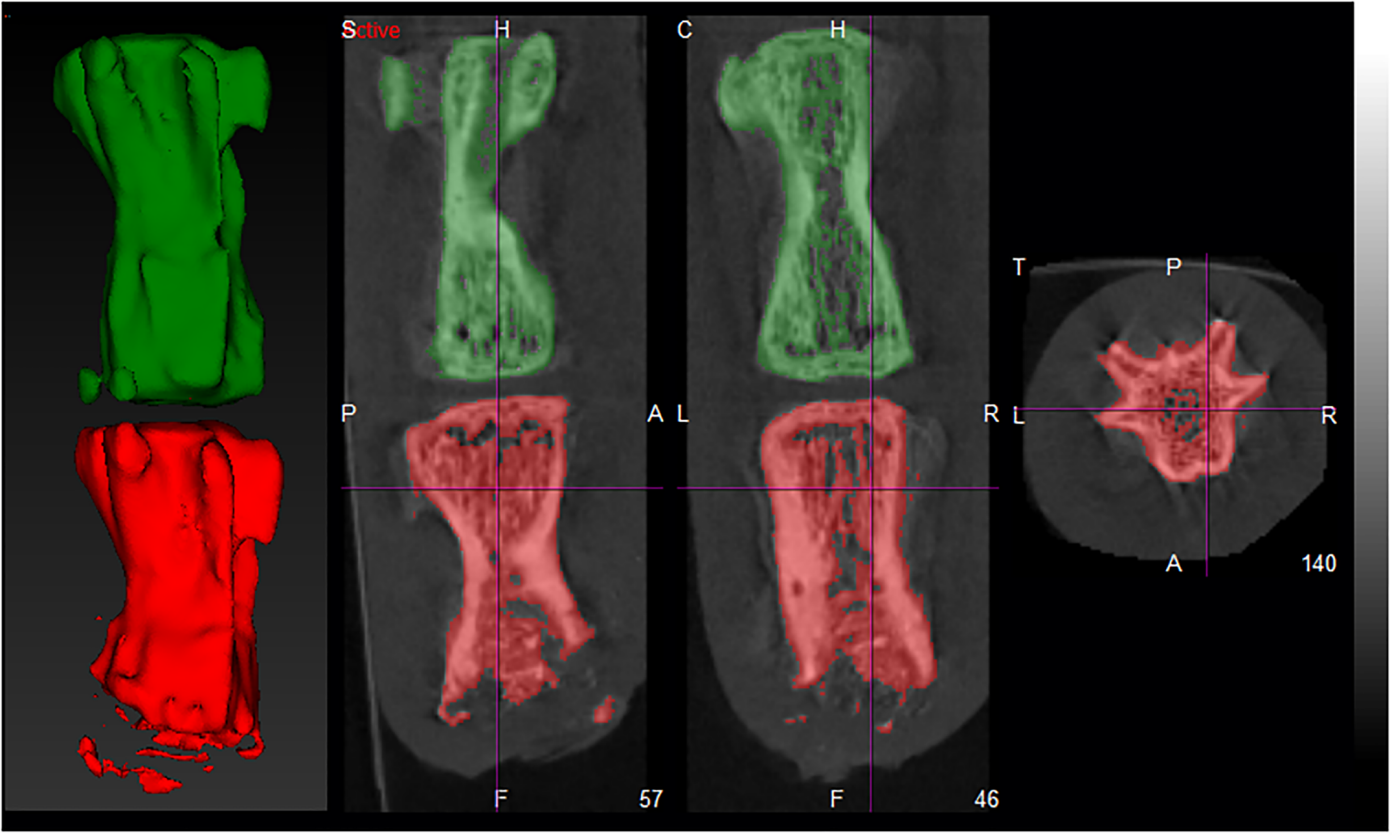
Structure of *caudal vertebrae* of treated rats at starting point. The C5 *vertebra* (red colour) was treated and filled with a bone graft which was selected as VOI for evaluation of CT. The green coloured C4 *vertebrae* show the VOI of control.

**Fig 2:**
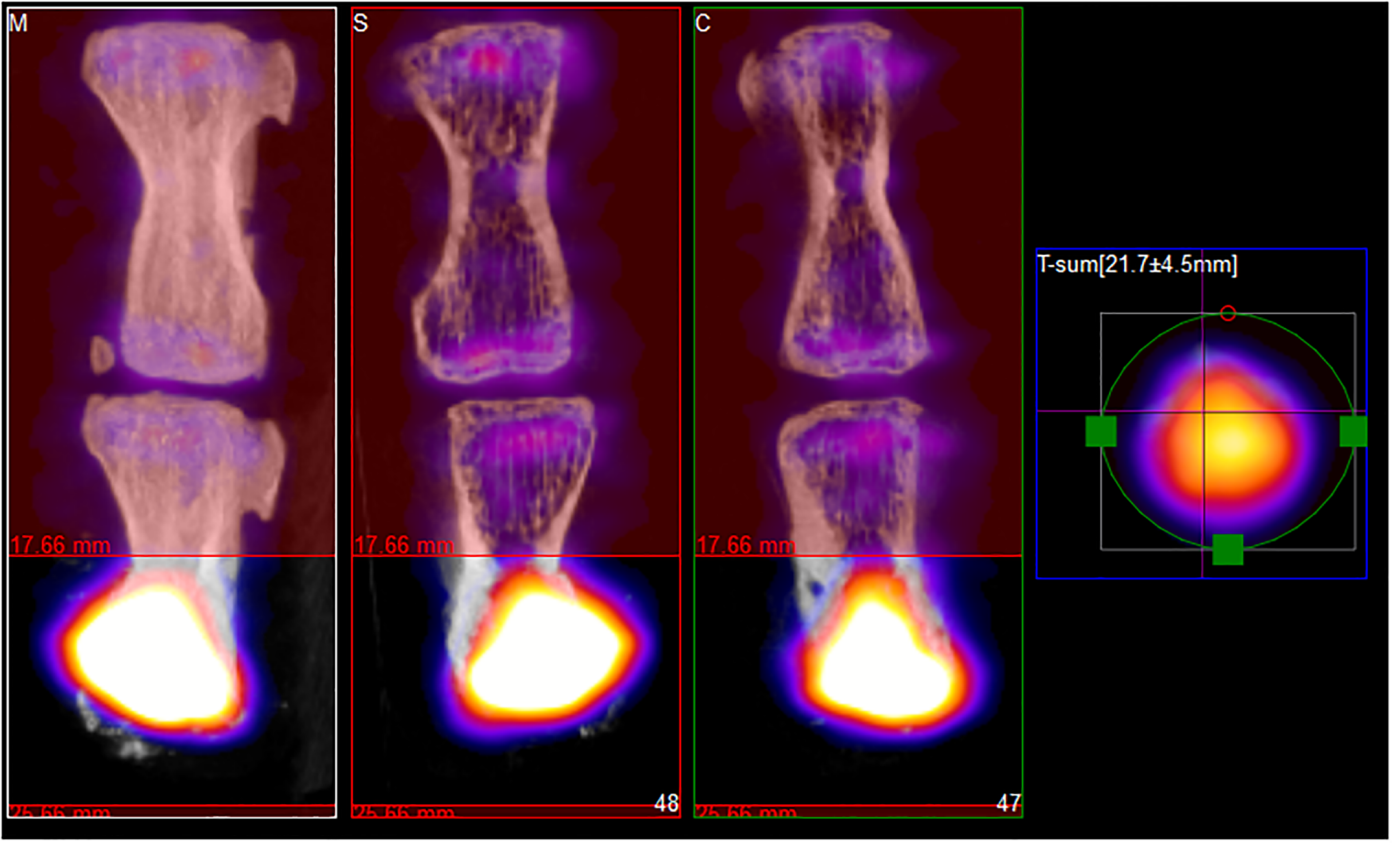
Tc-99m-MDP activity in *caudal vertebrae* of treated rats after eight weeks. The C5 *vertebrae* (down) were treated and filled with a bone graft which was selected as VOI in SPECT at eight weeks after surgery. The colour intensity shows the activity of Tc-99m-MDP in the last region of *vertebra*. The upper bones are C4 control *vertebrae*.

The mean opacity increased, while the mean Tc-99m-MDP activity decreased, in both of the groups and at both time points that are at 3 and 8 weeks following treatment. Fig 1 shows *vertebrae* at starting point while Fig 2 represents *vertebrae* after eight weeks. Fig 1 shows the exact anatomical position of VOI and Fig 2 the raw activity of Tc-99m-MDP only for the sake of illustration. Images showing the structure and the activity only represent similar measurements, since a visual comparison of the raw data derived from each acquisition does not provide enough information for proper quantitative evaluation. The CT is useful to detect structure (Fig 1), but the SPECT could provide the data of Tc-99m-MDP activity (Fig 2).

**Fig 3:**
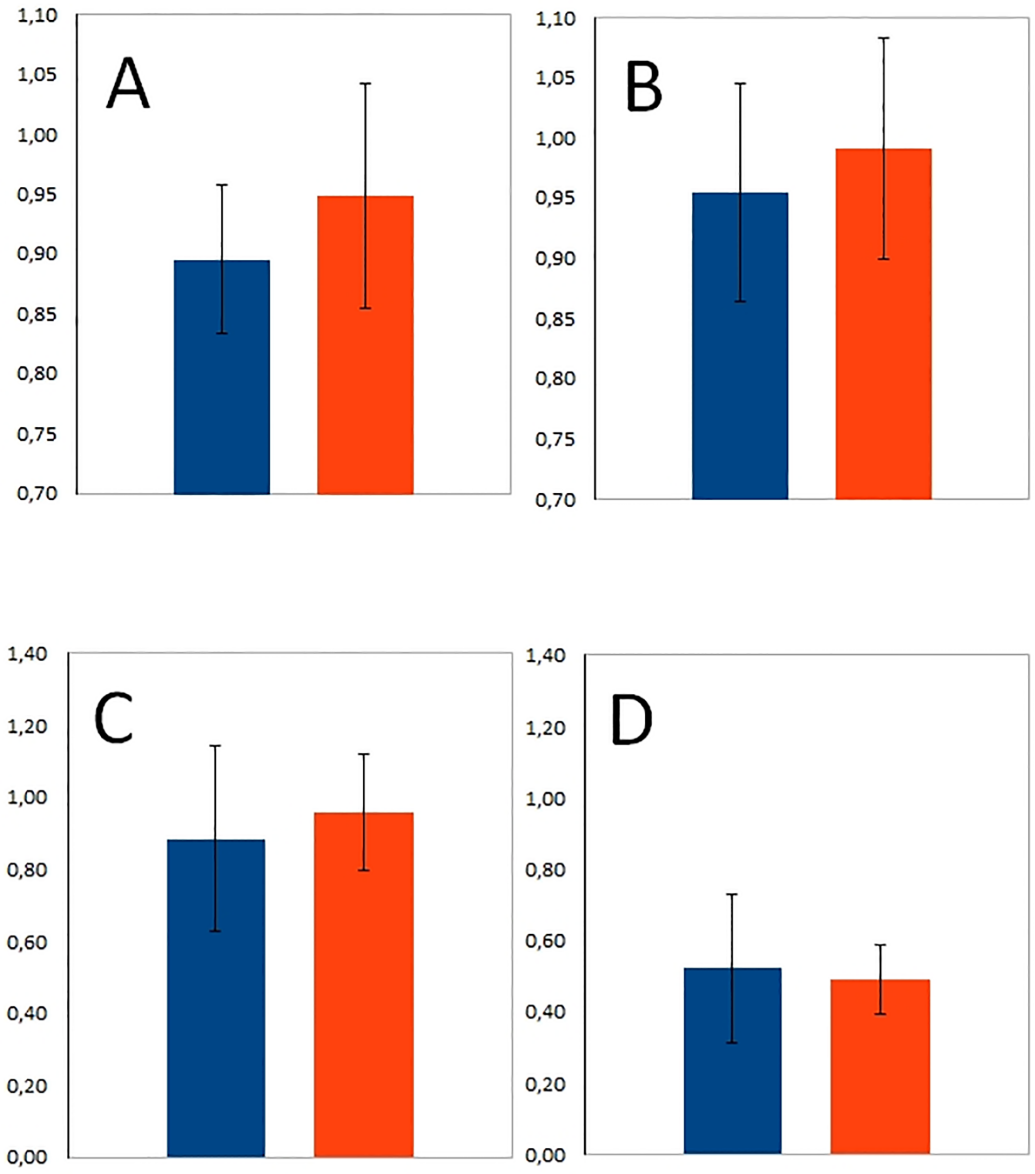
Mean opacity and mean Tc-99m-MDP activity. The figure represents the mean opacity (above) and the mean Tc-99m-MDP activity (below) of PMMA (blue) and HLBC (orange) at third weeks (A, C) and at eight weeks after surgery (B, D).

The difference between the PMMA and HLBC opacity values at the third and the eighth weeks were not significant (n = 4, p = 0.378) and (n = 4, p = 0.591), respectively (Fig 3A and 3B). Additionally, the difference between the PMMA and HLBC Tc-99m-MDP activity values at the third week were also not significant (n = 4, p = 0.651) (Fig 3C). Likewise, these differences at the eighth week (n = 4, p = 0.807) were also not significant (Fig 3D).

Only in the HLBC group, the activity of the mean of Tc-99m-MDP decreased significantly (n = 4, p = 0.002) starting from the third week until the eighth week (Fig 3C and 3D).

**Fig 4:**
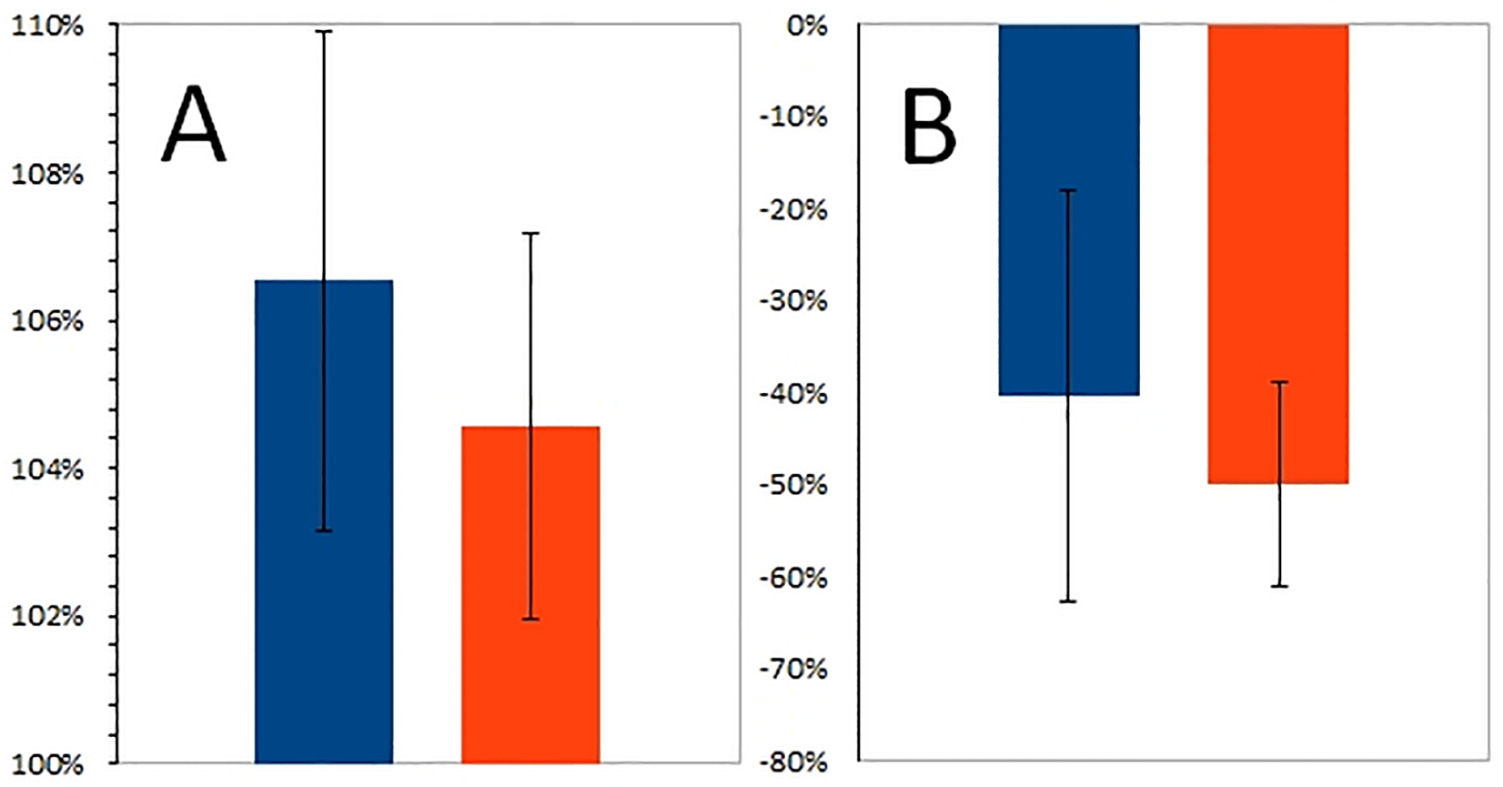
Change ratio %. Change ratio % from third week to eighth week after surgery of mean opacity increase (A) and mean Tc-99m-MDP activity decrease % (B) for PMMA (blue) and HLBC (orange) groups.

The mean opacity change in PMMA group compared to the same parameter of HLBC group was not significantly different (n = 4, p = 0.395) (Fig 4A). Similarly, the mean activity change of Tc-99m-MDP in PMMA and HLBC groups was not significant (n = 4, p = 0.468) (Fig 4B).

**Fig 5:**
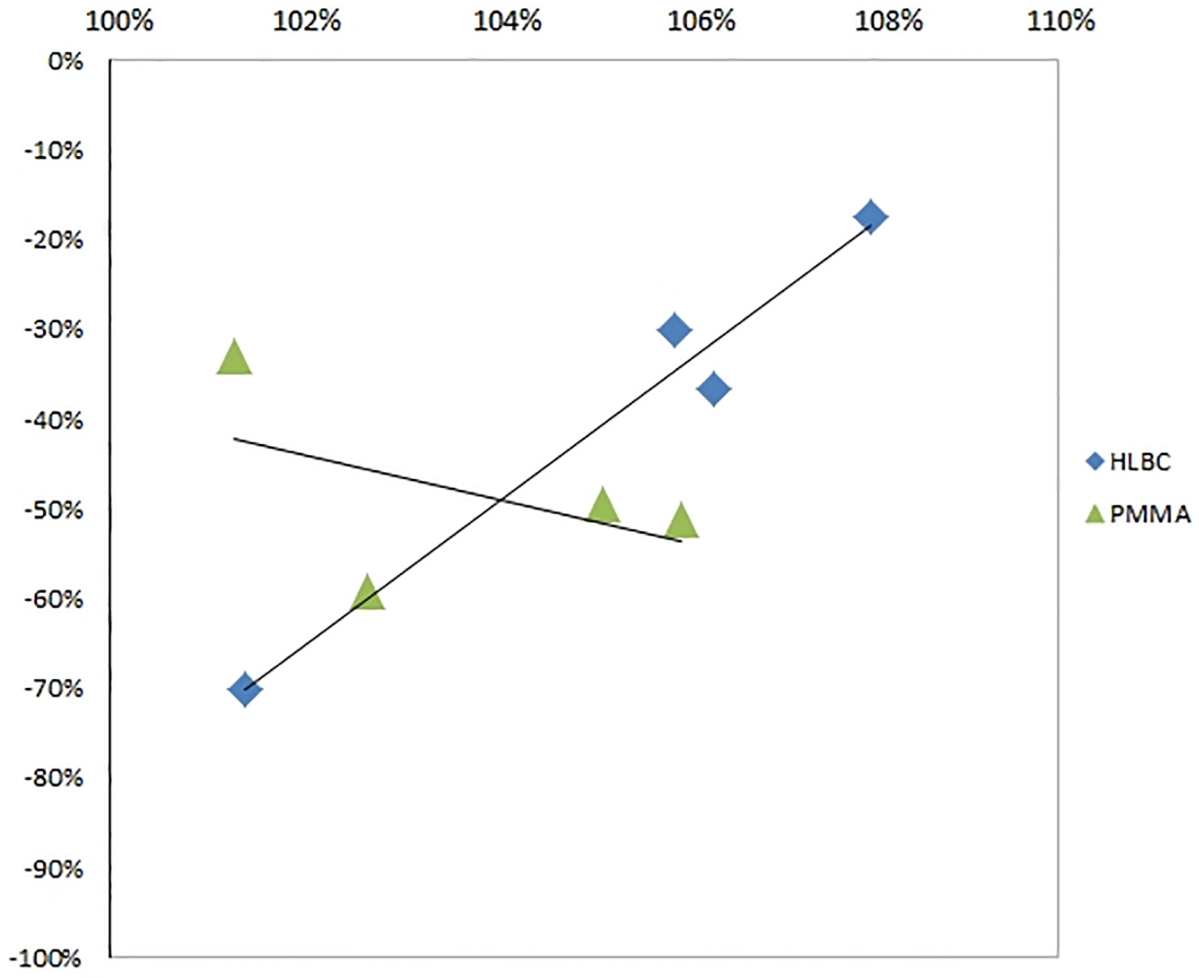
Linear regression. The ratio of opacity increasing % and the ratio of Tc-99m-MDP activity decreasing % from 3rd week after surgery to 8th week after surgery values for each individual animal in PMMA (triangle) and HLBC (diamond) groups were determined. A linear regression analysis was carried out.

Performing linear regression analysis (Fig 5), a strong positive correlation was found in HLBC group comparing the increase of bone opacity and decrease of Tc-99m-MDP activity variables (r = 0.772, p = 0.012).

In case of the PMMA treated group, medium negative correlation was found between these two variables (r = 0.395, p = 0.605).

With exception to one rat from each group, autopsy did not reveal any pathological condition including inflammation. In both groups the excluded rats have had inflammation.

## Discussion

The mean opacity increase of examined voxels in both groups between 3 and 8 weeks after surgery indicates the progress of bone tissue mineralization. The bone formation process was enhanced either by PMMA or HLBC, respectively (Fig 3A and 3B). Most of the grafts generally provided template to guide the repairing tissue [1, 25]. Thus, autonomic healing is achievable in PMMA bone cement brand [26]. It has to be noted that the mean opacity values of the two experimental groups, could not be distinguished from each other in a statistically significant manner. These indistinguishable values were obtained despite the fact that normalization of C5 vertebrae to intact C4 vertebrae and filtration of HU values below 1400 HU measures were undertaken (Fig 3A and 3B). Still, the applied bone graft attributions may cause slightly increase of the mean opacity of HLBC group when compared to PMME group [22]. Indeed, in MC3T3-E1 cell culture, the PMMA particles impaired cell proliferation and inhibited the expression of *RUNX2* and *DLX5* genes in a dose-dependent manner [27].

The HLBC enhanced bone formation in a previous publication, similarly to PMMA [22]. This was highlighted in specific Tc-99m-MDP uptake profile of both groups starting at the third week until eighth week after surgery. In the PMMA group, the Tc-99m-MDP uptake was slightly decreased, whereas, a strong decrease in HLBC group was obtained in a statistically significant manner (Fig 3C and 3D).

The increase of mean opacity ratio from third week to eighth week following surgery was stronger in the PMMA group than in the HLBC group (Fig 4A). However, in previous publications it was indicated that the PMMA enhanced bone formation less exquisitely and it was delayed when compared to HLBC's bone formation enhancement [28, 29]. Indeed, the low mean opacity value in the PMMA group at third week after surgery was reflected in the mean opacity ratio (Figs 3A and 4A). This result constitutes a spectacular example of advantages of multimodal imaging, since the aforementioned data are in concordance with Tc-99m-MDP uptake data. This uptake represents functional information that might highlight the features of bone formation process (Fig 4B). Thus, the change in the ratio by itself could not be used as a reliable descriptive parameter for bone formation, especially with such a low sample number as was the case in this experiment. In this investigational set-up, Tc-99m-MDP uptake ratio was informative in regard to the osteoblast activity [22] (Fig 4B). However, pathologic conditions such as neoplasticity, hormonal changes, inflammation, ischemia, may cause abnormal Tc-99m MDP uptake in soft-tissues, thereby, limiting the sensitivity of this method with false positive results [30].

Multimodal Tc-99m-MDP NanoSPECT/CT imaging utilizing radiomics evaluation has elucidated the decreasing Tc-99m-MDP uptake in relation to bone opacity change increase (Figs 4 and 5). The results of linear regression analysis pointed out the discrepancy between the examined group trends. The variables of PMMA group showed negative, while HLBC group’s revealed positive correlation (Fig 5).

Clearly, the PMMA lacks both osteoinductive and osteoconductive mechanisms [31]. Consequently, in the PMMA group, th osteoblast activity indicated a healing process showing a medium negative correlation with the bone density.

However, the HLBC showed a strong positive significant correlation between the increased opacity ratio and the decreased Tc-99m MDP activity. Probably because revascularization of the cancellous autografts takes place earlier than the cortical grafts’, as early as the fifth day following implantation, due to their porous architecture [28, 29]. In addition, in the HLBC group the creeping-substitution mechanism has enhanced vascularization and albumin-coated surface promoted osseointegration [24, 32].

Novelty of our method was that we did not only measure the bone opacity and the activity of a radioactive isotope, which parameters provide conventional data about density, formation and healing of bone, but we calculated linear regression from the opacity and the activity. The advantage of our method is that we could distinguish different bone grafts by healing efficacy which was impossible to achieve by existing techniques. The other advantage is that there is no additional cost since existing SPECT/X-ray CT instruments can be used without modification. The only disadvantage is that we needed to calculate an extra linear regression.

## Conclusions

The examined bone grafts have enhanced the bone mineralization process, as revealed by X-ray CT. Despite of normalization of the attenuation values of the C5 vertebra to the intact C4 vertebra, and the filtration of the opacity below 1400 HU, the difference between PMMA and HLBC groups was not statistically significant. The common attributions of the examined bone graft materials underpin a bone formation enhancer effect.

Using NanoSPECT, the decreased Tc-99m MDP activity was shown to be significant in the HLBC group between the third and eighth weeks following a surgery. With a linear regression analysis, the decrease in osteoblast activity ratio related to the increase in opacity ratio was examined. In the PMMA group, negative correlation was obtained, contrary to the results in the HLBC group.

The applied experimental set-up is cost-effective, presents quick and quantitative results and reduced the use of animals, in coherence with the European Union (EU) directive 2010/63/EU on the protection of animals [23]. This novel multimodal Tc-99m-MDP NanoSPECT/CT radiomics approach may be useful to compare several experimental bone replacement materials in preclinical studies. In addition, tracking of clinical bone formation can be established based on the results of this study. The presented multimodal *in vivo* imaging may support the optimal therapeutic strategy not only in correlation to bone healing, bone grafting and bone replacement, but also to the advancement of prognosis of bone diseases.

## Supporting information

S1 Table Raw data of opacity and activity. (XLSX)
